# Interaction of microtubules and actin during the post-fusion phase of exocytosis

**DOI:** 10.1038/s41598-019-47741-0

**Published:** 2019-08-19

**Authors:** M. Tabitha Müller, Rebekka Schempp, Anngrit Lutz, Tatiana Felder, Edward Felder, Pika Miklavc

**Affiliations:** 1 Institute of General Physiology, Ulm University, Albert-Einstein Allee 11, 89081 Ulm, Germany; 2 School of Environment and Life Sciences, University of Salford, The Crescent, M54WT Salford, United Kingdom

**Keywords:** Actin, Microtubules, Membrane fusion, Secretion, Exocytosis

## Abstract

Exocytosis is the intracellular trafficking step where a secretory vesicle fuses with the plasma membrane to release vesicle content. Actin and microtubules both play a role in exocytosis; however, their interplay is not understood. Here we study the interaction of actin and microtubules during exocytosis in lung alveolar type II (ATII) cells that secrete surfactant from large secretory vesicles. Surfactant extrusion is facilitated by an actin coat that forms on the vesicle shortly after fusion pore opening. Actin coat compression allows hydrophobic surfactant to be released from the vesicle. We show that microtubules are localized close to actin coats and stay close to the coats during their compression. Inhibition of microtubule polymerization by colchicine and nocodazole affected the kinetics of actin coat formation and the extent of actin polymerisation on fused vesicles. In addition, microtubule and actin cross-linking protein IQGAP1 localized to fused secretory vesicles and IQGAP1 silencing influenced actin polymerisation after vesicle fusion. This study demonstrates that microtubules can influence actin coat formation and actin polymerization on secretory vesicles during exocytosis.

## Introduction

Cellular secretion via regulated exocytosis is pivotally influenced by cell cytoskeleton. Microtubules and actin filaments provide tracks for secretory vesicle transport to the site of exocytosis^1–5^. Cortical actin presents a barrier for exocytosis and has to be remodelled to allow fusion between the vesicle and plasma membrane^6–9^. Formation of actin coat on fused secretory vesicles is essential for efficient extrusion of poorly soluble vesicular material^10–16^, stabilisation of fused vesicles^17,18^, and facilitation of compensatory endocytosis^19,20^.

Emerging evidence suggests that actin and microtubules can interact with each other^21–24^. Interaction between microtubules and actin filaments has been demonstrated in cell migration^25–27^, in elongation of neuronal protrusions^28–32^ and in formation of immunological synapses^33,34^. Microtubule-depolymerising agents were shown to increase actin polymerisation and formation of stress fibres^35–38^ most likely via activation of Rho family GTPases^23,39,40^. *In-vitro* experiments demonstrated that actin and microtubule cross-linking proteins can induce microtubule growth along actin fibres as well as actin filament elongation parallel to microtubules^41,42^. Actin and microtubule crosslinking proteins were suggested to connect both cytoskeletal networks and act as mediators in signalling cascades to control cytoskeletal remodelling^23^. One such protein is IQGAP1, which binds to actin directly^43^ and to microtubules indirectly via CLIP170^44^. IQGAP1 acts as a scaffold for proteins that regulate cytoskeletal remodelling^45–47^ and binds to small GTPases^48^ that are involved in regulation of actin and microtubule remodelling^23,40,49^. Actin nucleation factors N-WASP and mDia also directly bind to IQGAP1^50,51^.

Although experimental evidence suggests that actin and microtubules can interact with each other, the nature and significance of this interaction during exocytosis is not clear. We addressed this question in surfactant-secreting primary alveolar type II (ATII) cells. The large size of secretory vesicles (>1 µm) and slow fusion kinetics allow detection of individual vesicle fusion events^52,53^ and measurement of cytoskeletal remodelling^12,54^ using live-cell fluorescence microscopy. Fused vesicles in ATII cells acquire an actin coat, which is necessary for vesicle content extrusion^12,13,54^. Actin coat facilitates vesicle content release also in endothelial cells^15^, salivary gland cells^10,11^ and chromaffin cells^55^. The process of actin coat assembly involves de-novo actin polymerisation, mediated by Rho GTPases and formins in ATII cells^12^. In other cell models, the formation of actin coat was described to depend on formin or Arp2/3 nucleation factors^10,56^. The molecular mechanisms of actin coat polymerisation resemble the formation of cytokinetic ring during cell division and actin cup during phagocytosis^57^. In ATII cells, vesicle content extrusion is facilitated by actin coat contractility, which is visible as shrinkage of actin ring in epifluorescence microscopy^12,13,54^. The contraction of actin coats was shown to be partially mediated by myosin II^13,58^ and partially by the interaction between actin, actin depolymerising protein cofilin and actin crosslinking protein actinin^13^. A similar mechanism has also been described for cytokinetic ring contraction^59^. Interestingly, microtubules are involved in formation of cytokinetic ring^60,61^ and the phagocytic cup^62^. It is not known if microtubules are also involved in formation and function of exocytotic actin coats.

Here we show that microtubules in ATII cells localize near actin coats on fused secretory vesicles and stay close to the coats during coat compression. Inhibition of microtubule polymerisation with colchicine and nocodazole enhanced actin polymerisation on fused vesicles and influenced the kinetics of actin coat formation. Actin and microtubule crosslinking protein IQGAP1 and IQGAP1-associated protein CLIP170 localized to actin coats. IQGAP1 silencing decreased actin polymerisation on fused vesicles.

## Results

### Secretory vesicles in ATII cells are surrounded by microtubule network

**Figure 1:**
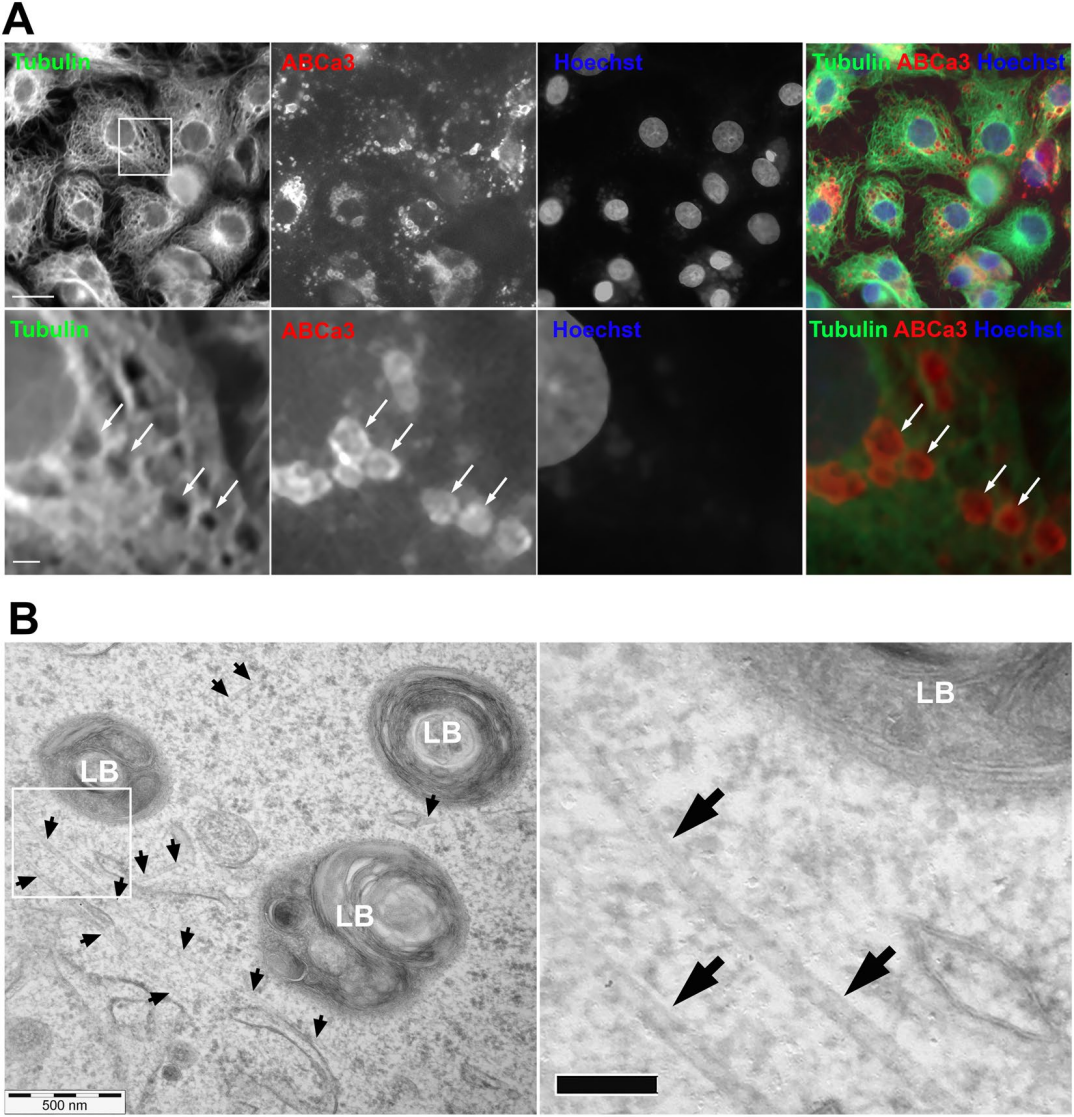
Secretory vesicles in primary isolated ATII cells are close to the microtubule network. (**A**) Primary isolated ATII cells were immunostained with anti-tubulin antibody and anti-ABCa3 antibody to visualize microtubules and secretory vesicles, respectively. ABCa3 is a transmembrane lipid transporter that localizes to lamellar body membrane and is a reliable marker for secretory vesicles in ATII cells. Cell nuclei were stained with Hoechst 33342. The boxed region in the top row is enlarged below. Arrows point at secretory vesicles. Scale bars: upper row 10 µm, lower row 1 µm. (**B**) Left: Transmission electron micrograph of ATII cell showing microtubules (arrows) close to secretory vesicles (lamellar bodies, LBs). The boxed region is enlarged on the right. Scale bar right: 100 nm.

To investigate whether microtubules influence actin coat formation and function in ATII cells we first explored the spatial relationship between the microtubule network and secretory vesicles using immunostaining and electron microscopy. Immunolabelling with α-tubulin and α-ABCa3 antibodies was used to visualize the microtubules and secretory vesicle membrane, respectively (Fig. 1A). Transmission electron microscopy showed that microtubules are localized close to secretory vesicles in ATII cells (Fig. 1B). Numerous microtubules were seen close to the vesicles on electron micrograph although the section thickness was approximately 70 nm. Quantification of microtubules on the vesicles less than 0.5 µm from the plasma membrane showed that 67.6% of vesicles (n = 34) had microtubules in their vicinity. There were 2.3 +/− 0.3 microtubules (mean +/− SEM) visible in the perimeter of 0.5 µm around the vesicle. Both methods suggest that secretory vesicles in ATII cells are localized near microtubule network.

### Microtubules are close to actin coats on fused secretory vesicles and remain close during actin coat compression

**Figure 2:**
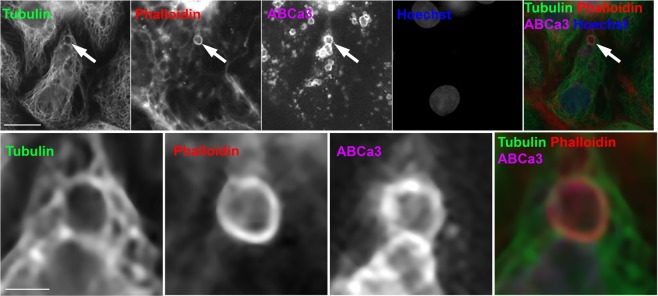
Microtubules localize close to actin coats on fused secretory vesicles. ATII cells were stimulated for secretion with ATP, fixed and stained with anti-tubulin antibody, anti-ABCa3 antibody and fluorescently labelled phalloidin to label microtubules, secretory vesicles and actin coats on secretory vesicles, respectively. Cell nuclei were labelled with Hoechst 33342. Arrows in the upper row point at fused secretory vesicle that is enlarged in the bottom row. Colour overlay shows the proximity between microtubules and actin coat. Scale bar above: 10 µm, below: 2 µm.

**Figure 3:**
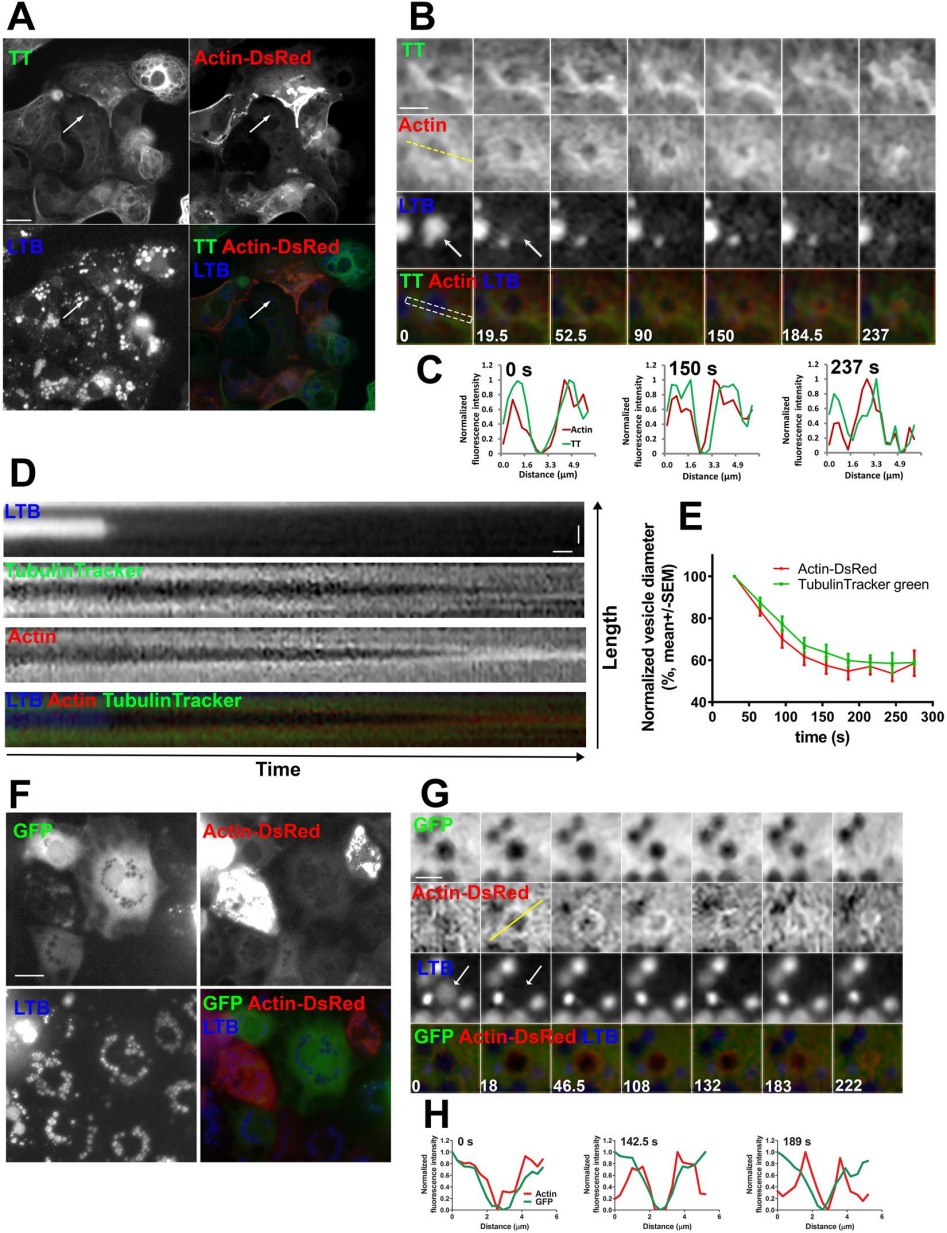
Compression of actin coats on fused secretory vesicles is accompanied by a change in microtubule localisation. (**A**) ATII cells transfected with actin-DsRed were stained with tubulin tracker green (TT) and LysoTracker blue (LTB) to label actin, microtubules and secretory vesicles, respectively. Arrow indicates a fusing secretory vesicle that is enlarged in (**B**). Scale bar: 10 µm. (**B**) Time lapse image sequence of TT, actin-DsRed and LTB fluorescence of a fusing vesicle. Numbers indicate time in seconds. Time = 0 is the last frame before vesicle fusion with the plasma membrane indicated by LTB fluorescence decrease (arrows). Vesicle fusion was followed by formation and compression of the actin coat. Microtubules translocated to the space that was occupied by the vesicle before actin coat compression. Fluorescence profiles on (**C**) were created using the yellow dashed line and the kymographs on D were created using the white rectangle. Scale bar: 2 µm. (**C**) Fluorescence intensity profiles of TT (green) and actin-DsRed (red) measured across the fusing vesicle (dashed yellow line on **B**). Numbers indicate time in seconds. (**D**) Kymographs of TT, actin-DsRed and LTB fluorescence were constructed using a region of interest across the fusing vesicle (dashed white rectangle on **B**). Actin coat compression was accompanied by translocation of microtubules. Scale bars: 10 s and 2 µm. (**E**) Compression of fused vesicles in cells transfected with actin-DsRed and stained with TT. Compression was measured as a decrease in vesicle diameter in actin-DsRed image sequence (red) and in TT image sequence (green). The lines indicate mean +/− SEM. N = 15 vesicles from 10 independent experiments and 3 cell isolations. (**F**) ATII cells were transfected with actin-DsRed and GFP control and stained with LysoTracker blue (LTB). Scale bar: 10 µm. (**G**) Time lapse image sequence of GFP, actin-DsRed and LTB fluorescence on a fusing vesicle. Numbers indicate time in seconds. Time = 0 is the last frame before vesicle fusion with the plasma membrane indicated by LTB fluorescence decrease (arrows). Yellow dashed line was used for creating fluorescence profiles on H. Scale bar: 2 µm. (**H**) Fluorescence intensity profiles of GFP and Actin-DsRed measured across the fusing vesicle (dashed yellow line on **G**). Numbers indicate time in seconds.

Next, we focused on localisation of microtubules relative to actin coats on fused vesicles. We stimulated ATII cells for secretion to induce actin coat formation and then used immunolabelling with anti-tubulin and anti-ABCa3 antibodies to visualize the microtubule network and secretory vesicles, respectively. Actin coats – characteristic for fused vesicles - were stained with alexa fluor 568 phalloidin. The overlay of the three channels confirmed that microtubules were in vicinity of actin coats after vesicle fusion with the plasma membrane (Fig. 2). Actin coats in ATII cells compress to provide the mechanical force for expulsion of secretory vesicle contents^13^. To investigate the dynamic interaction between actin coats and microtubule network, ATII cells were transfected with actin-DsRed and stained with tubulin tracker green (Fig. 3A). Secretory vesicles were stained with LysoTracker Blue (LTB; Fig. 3A) to detect the time of single exocytotic fusion events. LTB accumulates in acidic intracellular compartments such as lysosome-derived secretory vesicles in ATII cells and diffuses out of the vesicles after the fusion with the plasma membrane. Vesicle fusion therefore results in a rapid decrease in LTB fluorescence^52^. ATII cells were stimulated for secretion on a fluorescence microscope to generate time lapse image sequences (Fig. 3B). Microtubules remained in close apposition to actin coats throughout actin coat compression (Fig. 3B). Fluorescence intensity measurement across fusing vesicles (Fig. 3C, Supplementary Fig. 1) and fluorescence kymograph (Fig. 3D) showed proximity of both cytoskeletal networks during actin coat compression. To establish whether the movement of the compressing actin coat and adjacent microtubules occurred at the same time, we measured the compressing vesicle diameter in image sequences of actin-DsRed and tubulin tracker green (Fig. 3E). To show that increased tubulin fluorescence intensity around compressing actin coat was not due to accumulation of cytoplasmic components, the cells were transfected with GFP in addition to actin-DsRed and LTB staining (Fig. 3F). GFP transfected cells were stimulated for secretion on the fluorescence microscope to generate time-lapse images (Fig. 3G) and the fluorescence intensity was measured across the fusing vesicles (Fig. 3H, Supplementary Fig. 2). GFP is a cytoplasmic protein that is excluded from the vesicles, which results in a U shape of GFP fluorescence. The U shape became narrower during vesicle compression, however, the fluorescence peaks that were observed in tubulin tracker stained cells were not detected. To confirm the finding obtained with tubulin tracker staining, the cells were co-transfected with actin-GFP and tubulin-mRuby (Supplementary Fig. 3). In both sets of experiments microtubules remained close to actin coats during coat compression.

Microtubules are constantly growing and shrinking. To take this dynamics into consideration we transfected the cells with microtubule end-binding protein EB1-GFP (Supplementary Fig. 4 and Supplementary Movie 1), which labels microtubule plus ends^63,64^. We acquired fluorescence image sequence of EB1-GFP transfected cells and tracked EB1-GFP fluorescence over 200 frames (1.5 s/frame; 5 min) with Fiji software using Temporal-Color Code function to display EB1-GFP trajectories inside the cell (Supplementary Fig. 4A). To study the relation between microtubule trajectories and actin coats, ATII cells were co-transfected with EB1-GFP and actin-DsRed and stained with LTB (Supplementary Fig. 4B). Cells were stimulated for secretion on a microscope and fluorescence image sequence was acquired for all three channels. EB1-GFP labelled microtubule tips were close to compressing vesicles (Supplementary Fig. 4C) and moved along actin coats (Supplementary Fig. 4D–F).

### Colchicine and nocodazole, inhibitors of microtubule polymerisation, influence the kinetics of actin coat formation

After establishing a proximity between microtubules and actin coats we investigated whether the microtubules play a role for the post-fusion phase of exocytosis. Inhibition of microtubule polymerisation can induce the formation of actin stress fibres and focal adhesions^35–38^, therefore we measured whether inhibition of microtubule polymerisation with colchicine or nocodazole affects the polymerisation of actin on fused secretory vesicles.

**Figure 4:**
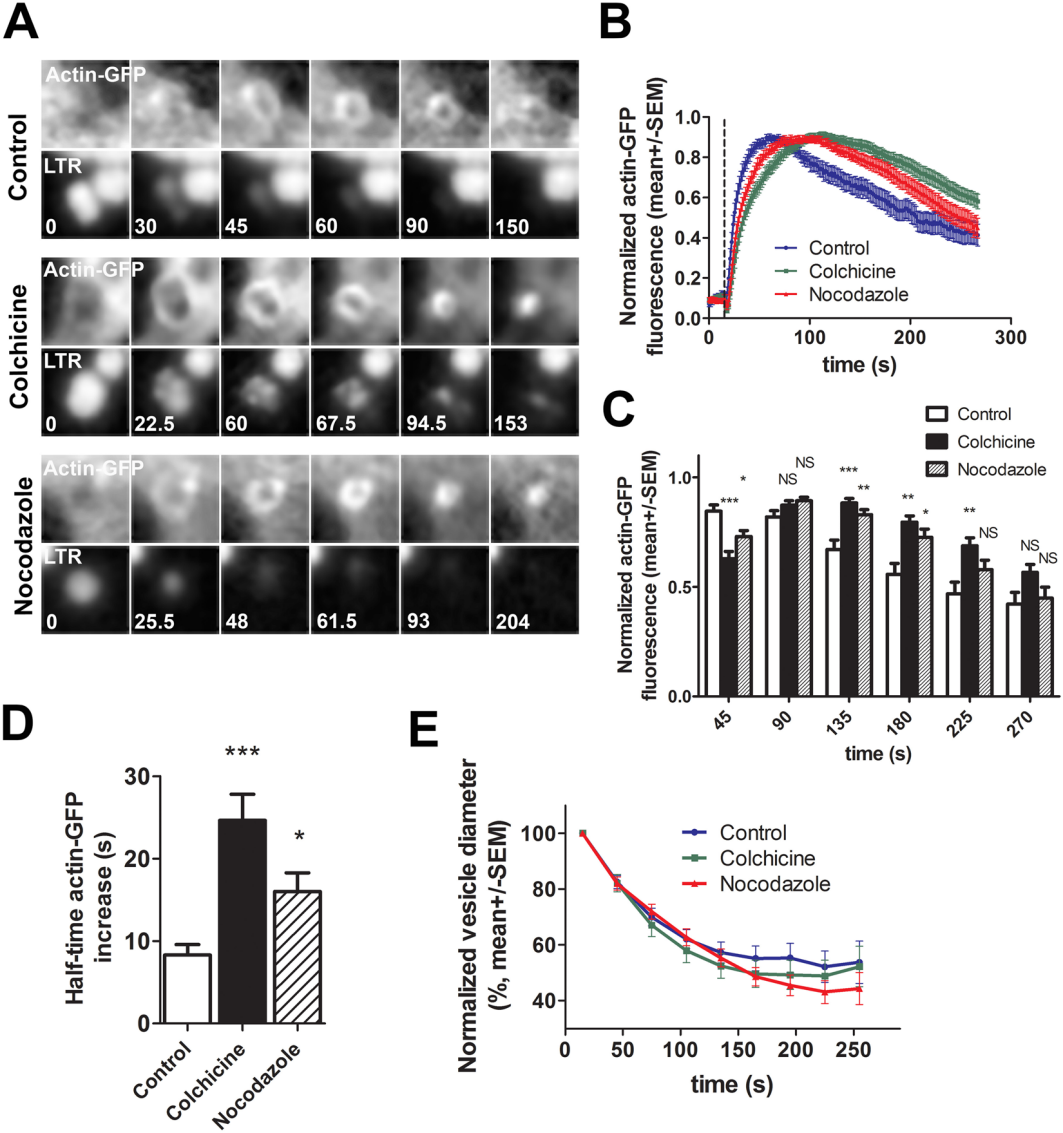
Cell treatment with colchicine and nocodazole changes the kinetics of actin coat formation. (**A**) ATII cells were transfected with actin-GFP and stained with LTR to detect actin coats on fused vesicles and the time point of exocytosis, respectively. Images show actin coat formation and compression in control, colchicine treated and nocodazole treated cells. Numbers indicate time in seconds, time 0 is the last frame before vesicle fusion. (**B**) Mean +/− SEM of normalized actin-GFP fluorescence intensity change on a circular region of interest surrounding a fusing secretory vesicle. N = 18 (control), 28 (colchicine) and 31 vesicles (nocodazole). All measured actin coats fully compressed during the experiment. The measurements are from 7 (control and nocodazole) and 10 independent experiments (colchicine) and 3 cell isolations. Dashed line indicates the time of vesicle fusion (t = 15 s). (**C**) Normalized GFP fluorescence intensity was measured in control, colchicine treated and nocodazole treated cells at selected timepoints on B. *P < 0.05; **P < 0.01; ***P < 0.001; NS: not significant; one-way ANOVA with Kruskal-Wallis test and Dunn’s multiple comparison test. (**D**) Half time of actin-GFP fluorescence increase during actin coat formation in control, colchicine treated and nocodazole treated cells. N = 18 (control), 28 (colchicine) and 31 (nocodazole). *P < 0.05; ***P < 0.001; one-way ANOVA with Kruskal-Wallis test and Dunn’s multiple comparison test. (**E**) Actin coat compression was analysed by measuring the diameter of the actin coat on actin-GFP fluorescence image sequence. Time = 0 is the time of vesicle fusion. The first measurement of actin coat diameter was 15 s after fusion. Compression of actin coat was not significantly different in control and in cells treated with colchicine or nocodazole (two-tailed t-test). Data are from 29 actin coats, 5 independent experiments and 3 cell isolations (control) and 28 actin coats, 4 independent experiments and 3 cell isolations (colchicine and nocodazole).

Immunostaining of ATII cells treated with either colchicine (50 µM for 3 h) or nocodazole (60 µM for 30 min) with anti-β-tubulin antibody confirmed that both treatments prevented microtubule polymerisation (Supplementary Fig. 5). We transfected ATII cells with actin-GFP to investigate actin coats and stained secretory vesicles with LysoTracker Red (LTR) to determine the time point of single vesicle fusion. Cells were treated with colchicine or nocodazole and stimulated for secretion under a fluorescence microscope to record the formation of actin coats on image series (Fig. 4A). Compressing actin coats in cells treated with colchicine or nocodazole reached the peak GFP fluorescence intensity significantly slower than those in control cells (Fig. 4B). Mean +/− SEM time of the peak fluorescence was 75.3 +/− 7.5 s after fusion for control, 112.4 +/− 6.8 s for colchicine and 103 +/− 6.9 s for nocodazole (p < 0.01 for colchicine and p < 0.05 for nocodazole; one-way ANOVA with Kruskal-Wallis test and Dunn’s multiple comparison test). The measurements were from 18 (control), 28 (colchicine) and 31 actin coats (nocodazole); 7 (control and nocodazole) and 10 independent experiments (colchicine); and 3 cell isolations. Normalized actin-GFP fluorescence on actin coats was compared in control and colchicine or nocodazole treated cells at different time points during actin coat formation and compression (Fig. 4C). Actin coat fluorescence intensity in cells treated with colchicine or nocodazole was significantly lower than in control 30 s after vesicle fusion (t = 45 s, Fig. 4C) and significantly higher than control 120 s, and 165 s after fusion (t = 135 and 180, Fig. 4C). Half-time of actin coat fluorescence increase measured by one-phase association fit was significantly higher in colchicine and nocodazole treated cells (24.6 +/− 1.3 s and 16 +/− 2.3 s; p < 0.001 and p < 0.05, respectively; Fig. 4D) than in control cells (8.3 +/− 1.3 s). One-way ANOVA with Kruskal-Wallis test and Dunn’s multiple comparison test were used for 18 (control), 28 (colchicine) and 31 (nocodazole) actin coats. To investigate if the differences in actin coat formation had an impact on actin coat function, we measured the normalized actin coat diameter during compression (Fig. 4E). There were no significant differences in the compression kinetics. Data are from 29 actin coats, 5 independent experiments and 3 cell isolations (control) and 28 actin coats, 4 independent experiments and 3 cell isolations (colchicine and nocodazole).

Together, these experiments suggest different kinetics of actin coat formation in colchicine-treated and in nocodazole-treated cells compared to control. However, changed kinetics did not significantly influence actin coat compression.

### Colchicine and nocodazole treatment increase actin polymerisation on actin coats

**Figure 5:**
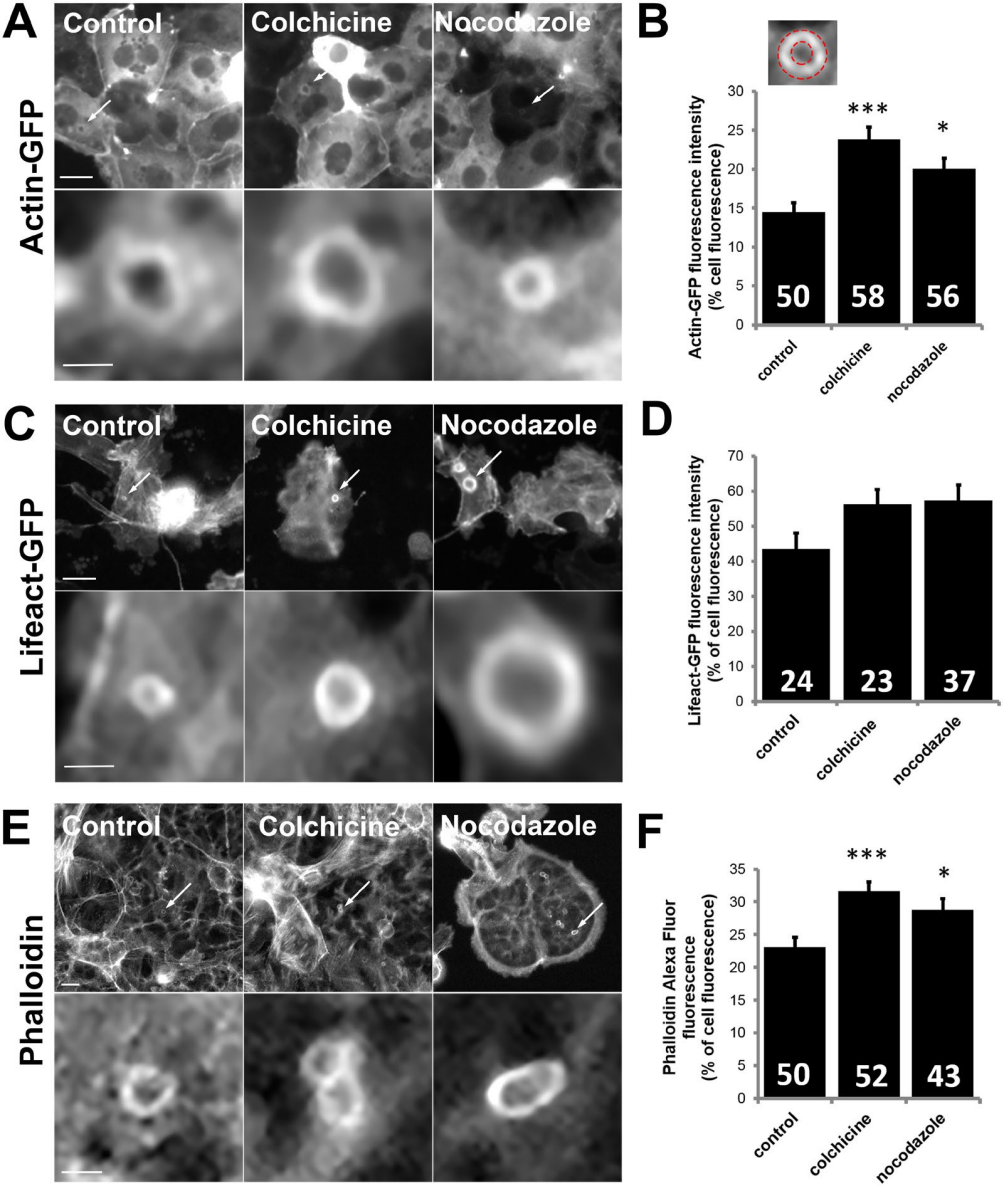
Cell treatment with colchicine and nocodazole influences the extent of actin polymerisation on fused secretory vesicles. (**A**) ATII cells transfected with actin-GFP were treated with colchicine or nocodazole and stimulated for secretion under the microscope. Exocytosis was detected with LTR. Arrows and inserts show actin coats on fused vesicles. Scale bar: 10 µm (above), 2 µm (below). (**B**) Actin-GFP fluorescence was measured in a ring-shaped region of interest on the actin coat (insert) and expressed as percent increased fluorescence intensity compared to cell cytoplasm fluorescence. Mean +/− SEM is shown and the numbers indicate the number of actin coats. Actin coats were from 5 (control) or 6 independent experiments (colchicine and nocodazole) and 3 cell isolations (***P < 0.001, *P < 0.05, one-way ANOVA with Tukey’s multiple comparison test). (**C**) ATII cells were transfected with lifeact-GFP, treated with colchicine or nocodazole and stimulated for secretion under the microscope to image actin coats (arrows and inserts). Exocytosis was detected with LTR. Scale bar: 10 µm (above), 2 µm (below). (**D**) Lifeact-GFP fluorescence was measured in a ring-shaped region of interest on the actin coat (as shown on 4B) and expressed as percent increased fluorescence intensity compared to cell cytoplasm fluorescence. Mean +/− SEM is shown and the numbers indicate the number of actin coats. Actin coats were from 4 independent experiments and 4 cell isolations (control), 8 independent experiments and 5 cell isolations (colchicine), and 9 independent experiments and 4 cell isolations (nocodazole). P = 0.07; one-way ANOVA with Tukey’s multiple comparison test. (**E**) ATII cells were treated with colchicine or nocodazole, stimulated for secretion, fixed and stained with alexa fluor 568 phalloidin. Actin coats on fused secretory vesicles (arrows) are enlarged below. Secretory vesicles were recognised by anti-ABCa3 staining (Supplementary Fig. 6). Scale bar: 10 µm (above), 2 µm (below). (**F**) Alexa fluor 568 phalloidin fluorescence was measured in a ring-shaped region of interest on the actin coat (as shown on 4B) and expressed as percent increased fluorescence intensity compared to cell cytoplasm fluorescence. Mean +/− SEM is shown and the numbers indicate the number of actin coats. The actin coats were from 5 independent experiments and 5 cell isolations (***P < 0.001, *P < 0.05, one-way ANOVA with Kruskal-Wallis test and Dunn’s multiple comparison test).

Next, we investigated if microtubule depolymerization with colchicine or nocodazole affects the extent of actin coat assembly on fused vesicles. ATII cells were transfected with actin-GFP or lifeact-GFP to label actin coats. The GFP fluorescence intensity of actin coats was measured to estimate the level of actin polymerisation (Fig. 5A,C, respectively). Cells were transfected with actin-DsRed 24 hours before experiment and secretory vesicles were labelled with LTR to detect the time of individual fusion events. The cells were treated with either colchicine or nocodazole and stimulated for secretion on a fluorescence microscope. We estimated the extent of actin polymerisation on fused vesicles by measuring actin coat fluorescence intensity relative to cell cytoplasm fluorescence intensity. Fluorescence of fully formed actin coats was measured on a ring-shaped region of interest before coat compression (Fig. 5B) and expressed as percent increase from the cell cytoplasm fluorescence (Fig. 5B,D; see also Materials and Methods). An additional approach to estimate the extent of actin coat polymerisation was to label actin coats in fixed cells. The cells were treated with either colchicine or nocodazole, stimulated for secretion with ATP, fixed, immunostained for ABCa3 and alexa fluor 568 phalloidin and imaged using identical conditions and settings (Fig. 5E and Supplementary Fig. 6). Actin coat fluorescence intensity relative to cell cytoplasm fluorescence was measured as above (Fig. 5F).

All three approaches demonstrated a marked increase in actin coat fluorescence after treatment with colchicine and nocodazole compared to control cells. In actin-GFP transfected cells actin coat fluorescence intensity increase was significantly lower in control (14.48 +/− 1.22%; n = 50) than in cells treated with colchicine (23.82 +/− 1.60%; n = 58; P < 0.001) or nocodazole (20.03 +/− 1.35%; n = 56; P < 0.05, one-way ANOVA with Tukey’s multiple comparison test; Fig. 5B). Likewise, in lifeact-GFP transfected cells actin coat fluorescence intensity increase was lower in control (43.45 +/− 4.49%; n = 24) than in cells treated with colchicine (56.15 +/− 4.19%; n = 23) or nocodazole (57.27 +/− 4.40%; n = 37, P = 0.07; one-way ANOVA with Tukey’s multiple comparison test; Fig. 5D). In cells stained with alexa fluor 568 phalloidin the control actin coat fluorescence increase (23.09 +/− 1.50%; n = 50) was significantly lower than in cells treated with colchicine (31.59 +/− 1.42%; n = 52; P < 0.001) or nocodazole (28.82 +/− 1.64%; n = 43; P < 0.05, one-way ANOVA with Kruskal-Wallis test and Dunn’s multiple comparison test; Fig. 5F). Therefore, it is likely that inhibition of microtubule polymerisation with colchicine or nocodazole induced stronger actin polymerisation on fused secretory vesicles.

### Actin and microtubule cross-linking protein IQGAP1 localizes to actin coats on fused secretory vesicles

**Figure 6:**
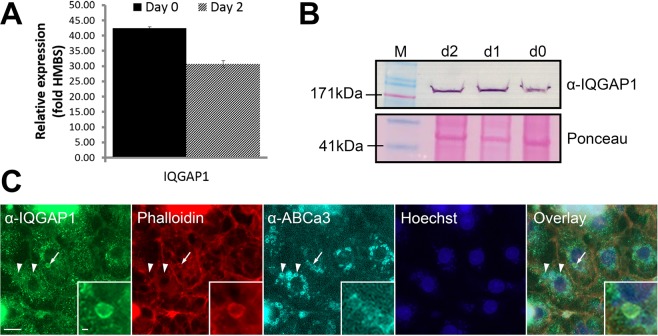
Actin and microtubule associated protein IQGAP1 localizes to actin coats on fused secretory vesicles. (**A**) IQGAP1 expression in ATII cells was measured with RT-PCR relative to the expression of the housekeeping gene *HMBS* immediately after isolation (day 0) and after 2 days of cell culture (day 2). (**B**) Western blot with α-IQGAP1 antibody with freshly isolated ATII cells (d0), after 1 day of culture (d1) and after 2 days of culture (d2). M = Molecular weight marker. Ponceau S staining was used as control for equal loading. Full-length gel is shown on Supplementary Fig. 7. (**C**) ATII cells were stimulated for secretion with ATP, fixed and immunolabelled for IQGAP1 and ABCa3. Actin was labelled with alexa fluor 568 phalloidin and cell nuclei with Hoechst 33342. Fused secretory vesicles (arrow and inserts) were identified by the presence of the actin coat. Overlay shows colocalization between IQGAP1, actin coat and secretory vesicle. Arrowheads: non-fused vesicles; scale bar: 10 µm, insert: 2 µm.

To explore if actin and microtubule cross-linking proteins are involved in the interaction between microtubules and actin coat, we investigated the expression and localisation of IQGAP1, which is involved in regulation of actin and microtubule dynamics^23^. Real-time PCR (RT-PCR) and western blot showed that IQGAP1 is expressed in ATII cells (Fig. 6A,B and Supplementary Fig. 7). In cells immunolabeled with α-IQGAP1 antibody, α-ABCa3 antibody and alexa fluor 568 phalloidin, IQGAP1 co-localized with actin coats on fused secretory vesicles (Fig. 6C). IQGAP1 was not visible on non-fused vesicles, so it is likely that it translocates to vesicles after exocytosis.

**Figure 7:**
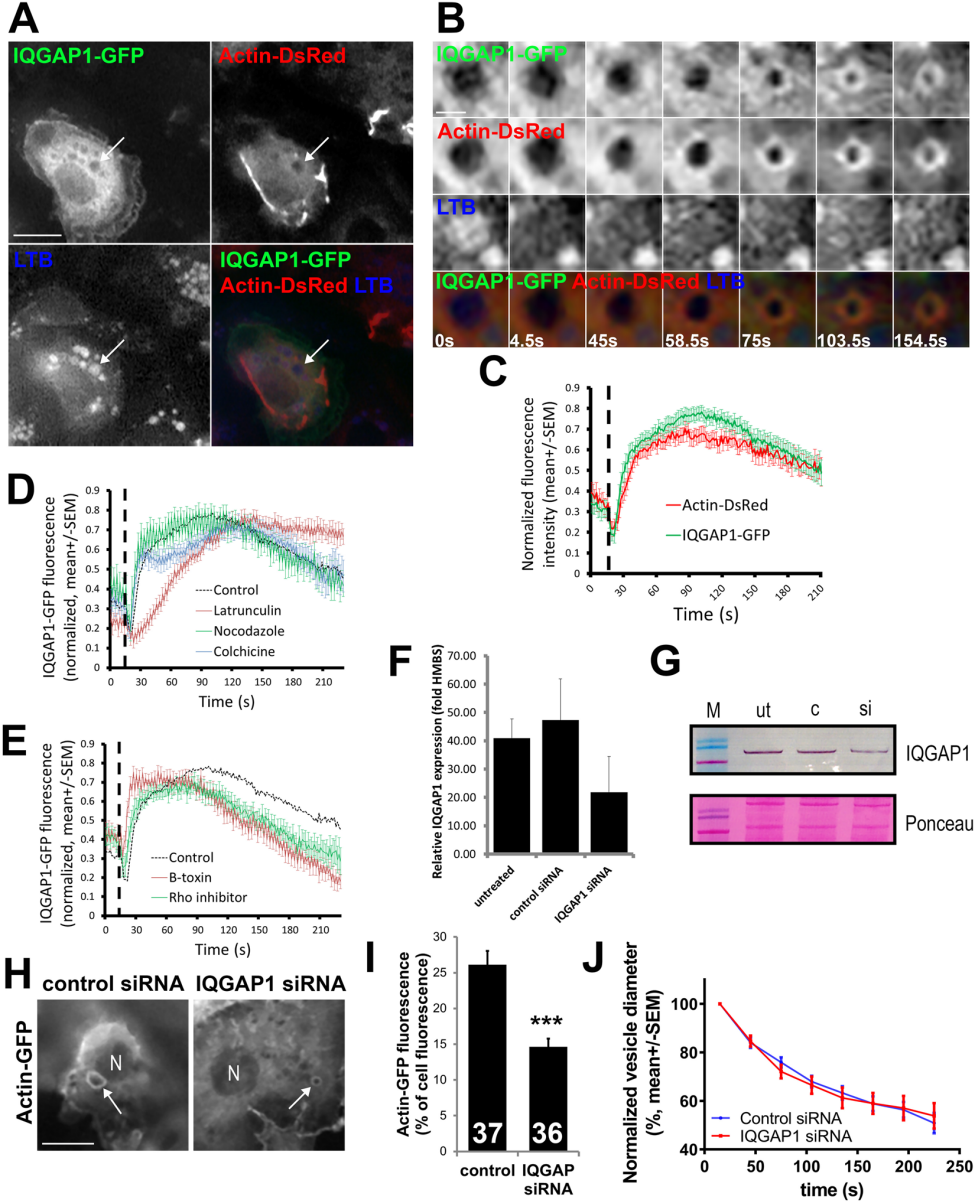
IQGAP1 translocates to secretory vesicles after fusion whereas IQGAP1 silencing decreased actin-GFP fluorescence on actin coats. (**A**) ATII cells were transfected with IQGAP1-GFP, actin-DsRed and stained with LTB. Arrow: fusing vesicle enlarged on B; scale bar: 10 µm. (**B**) Changes in IQGAP1-GFP, actin-DsRed and LTB fluorescence during exocytosis. Numbers indicate time; scale bar: 2 µm. (**C**) IQGAP1-GFP and actin-DsRed fluorescence intensity change on fusing vesicles (29 vesicles, 12 experiments and 5 cell isolations). Dashed lines on C, D, E: time of fusion. (**D**) IQGAP1-GFP fluorescence on fusing vesicles in cells treated with latrunculin, colchicine or nocodazole (31 vesicles, 8 experiments and 4 cell isolations (latrunculin); 28 vesicles, 7 experiments and 3 cell isolations (colchicine); and 21 vesicles, 11 experiments and 5 cell isolations (nocodazole)). The dotted line on D and E shows control IQGAP1-GFP fluorescence. (**E**) IQGAP1-GFP fluorescence on fusing vesicles in cells treated with B toxin or Rho-GTPase inhibitor (38 vesicles, 18 experiments and 3 cell isolations (B toxin); 24 vesicles, 8 experiments and 3 cell isolations (Rho inhibitor)). (**F**) ATII cells were transfected with IQGAP1-silencing siRNA or control siRNA and relative IQGAP1 expression was measured with RT-PCR (mean +/− SD, 3 cell isolations). (**G**) Immunoblotting with α-IQGAP1 three days after cell isolation and transfection with IQGAP1-silencing siRNA (si) or control siRNA (c). Ut: untreated control. Full-length gel is on Supplementary Fig. 8. (**H**) Cells transfected with IQGAP1-silencing siRNA or control siRNA were co-transfected with actin-GFP, stimulated for exocytosis and imaged for actin coat formation. N: nucleus; arrows: actin coats; scale bar: 10 µm. (**I**) Actin-GFP fluorescence on actin coats was measured as described on Fig. 4. Numbers indicate the number of measured actin coats (9 experiments and 3 cell isolations (control siRNA), 7 experiments and 3 cell isolations (IQGAP1 siRNA)). ***P < 0.001; two-tailed t-test. (**J**) Compression of actin coats in cells transfected with IQGAP1 siRNA was measured as a decrease in actin coat diameter (16 vesicles, 8 independent experiments and 3 cell isolations (control siRNA); 19 vesicles, 5 independent experiments and 3 cell isolations (IQGAP1 siRNA)).

To further investigate IQGAP1 translocation to fused vesicles, we co-transfected cells with IQGAP1-GFP and actin-DsRed and stained the secretory vesicles with LTB (Fig. 7A). ATII cells were stimulated for secretion under the fluorescence microscope to record image sequences (1 frame/1.5 s; 5 min recordings) and visualize single vesicle fusion events in all three channels (Fig. 7B). The quantitative analysis of the fluorescence signal on the region of interest surrounding fusing vesicles demonstrates IQGAP1-GFP translocation to secretory vesicles after fusion (Fig. 7C).

To determine if IQGAP1-GFP translocation to fused vesicles was influenced by actin or by microtubules, we treated cells with either latrunculin or with colchicine or nocodazole to inhibit actin polymerisation and microtubule polymerisation, respectively. Treatment with latrunculin resulted in slower IQGAP1-GFP translocation, whereas treatment with colchicine or nocodazole did not have an affect (Fig. 7D). We further investigated whether IQGAP1-GFP translocation was dependent on Rho GTPases. Brandt *et al*. (2007) showed that IQGAP1 delivers formin nucleation factor Dia1 to the site of Rho-mediated actin nucleation^51^ and we have previously shown that active Rho localizes to fused secretory vesicles in ATII cells^13^. Inhibitor of small GTPases *Clostridium difficile* B toxin and inhibitor of Rho GTPases prevented actin coat formation^12^. However, here we found that neither B toxin nor Rho GTPase inhibitor prevented translocation of IQGAP1-GFP to fused vesicles (Fig. 7E). Together this suggests that IQGAP1-GFP translocation to fused secretory vesicles was independent of microtubules and actin Rho-GTPases and had a delayed peak by the absence of actin coat.

To investigate the function of IQGAP1 we used siRNA silencing. ATII cells were transfected with siRNA against IQGAP1 after cell isolation, harvested 3 days later and analysed for the efficiency of IQGAP1 silencing with RT-PCR and western blot (Fig. 7F,G and Supplementary Fig. 8). siRNA treated cells were also co-transfected with actin-GFP and stimulated for secretion under the fluorescence microscope. Fused vesicles in cells transfected with IQGAP1 siRNA acquired actin coats (Fig. 7H). However, the relative intensity of actin coat fluorescence in cells transfected with IQGAP1 siRNA (14.62 +/− 1.17%; n = 36) was significantly lower than actin coat fluorescence intensity in cells treated with control siRNA (26.12 +/− 1.93%; n = 37; P < 0.0001, two-tailed t-test; Fig. 7I). Actin coat compression in cells treated with IQGAP1 siRNA was not significantly different from cells transfected with control siRNA (Fig. 7J; two-tailed t-test).

**Figure 8:**
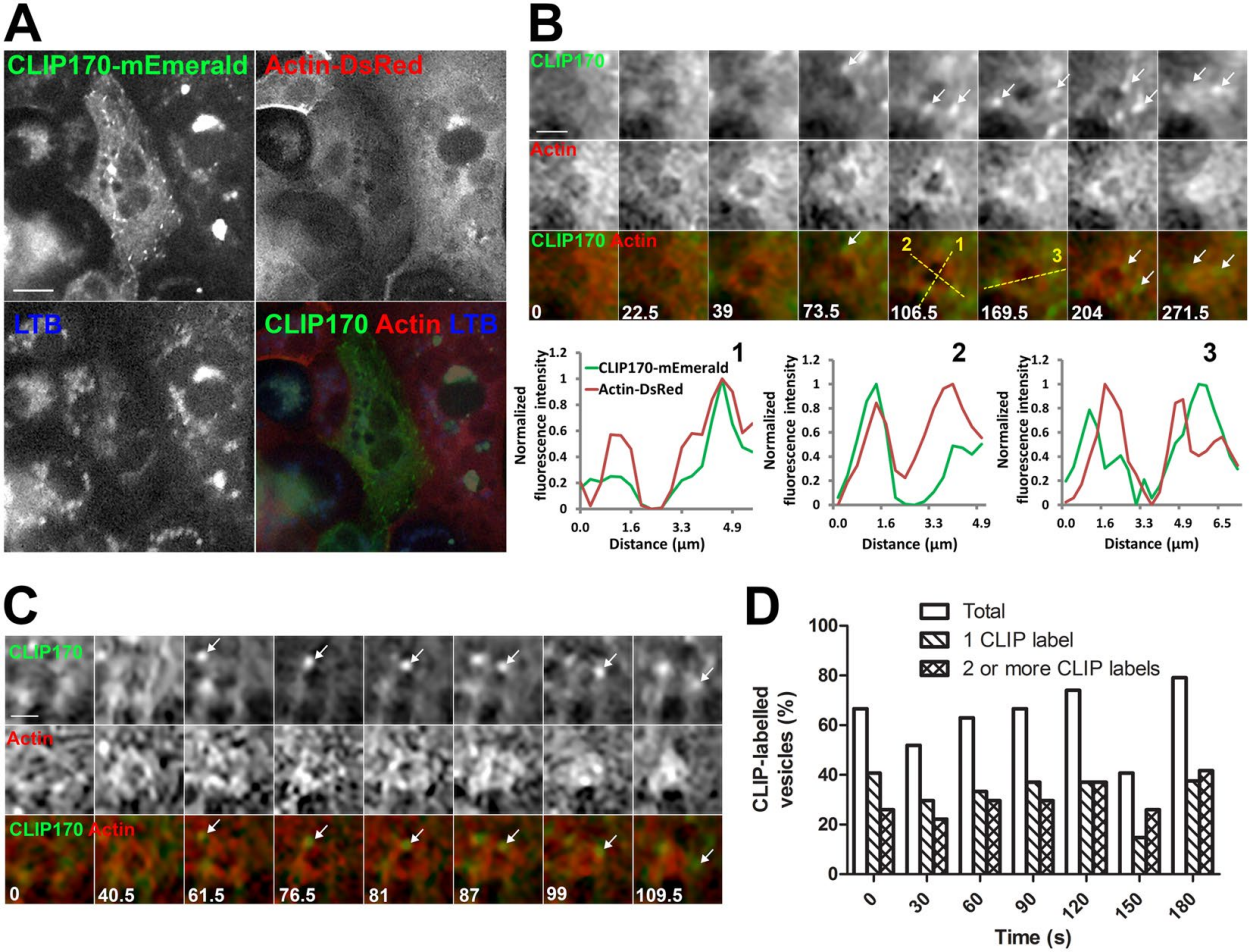
CLIP170-mEmerald-labelled microtubule tips localize close to actin coats. (**A**) ATII cells were co-transfected with CLIP170-mEmerald and actin-DsRed. Vesicles were stained with LTB to detect the time of fusion. Scale bar: 10 µm. (**B**) Image sequence of a fusing vesicle showing microtubule tip localisation (CLIP170-mEmerald) and actin coat formation (actin-DsRed). CLIP170-mEmerald-labelled microtubule growing ends were close to the fusing vesicle during actin coat compression (arrows). Time stamps indicate time in seconds; time = 0 is the last frame before fusion. Scale bar: 2 µm. Diagrams show the fluorescence intensity profile across the fusing vesicle at times and locations indicated by dashed lines 1–3. See Supplementary Movie 2 for the whole image sequence. (**C**) Image sequence of a fusing vesicle in a cell transfected with CLIP170-mEmerald (green) and actin-DsRed (red). Growth of one CLIP170-mEmerald-labelled microtubule tip along the compressing actin coat is followed over time (arrows). Time stamps indicate time in seconds; time = 0 is the last frame before fusion. Scale bar: 2 µm. (**D**) Microtubule tip localization to fusing secretory vesicles was quantified over time in cells co-transfected with CLIP170-mEmerald and actin-DsRed. CLIP-labelled tips were quantified on a region of interest around the actin coat at different time points on the image sequence. Time = 0 is the last time frame before fusion. Data were obtained from 27 vesicles, 14 independent experiments and 5 cell isolations.

IQGAP1 binds to microtubules indirectly via microtubule end-binding protein CLIP170. To better understand the relationship between IQGAP1, actin coats and microtubules we transfected ATII cells with CLIP170-mEmerald and actin-DsRed. Secretory vesicles were stained with LTB to detect the time of exocytosis (Fig. 8A and Supplementary Movie 2). CLIP170-mEmerald labelled growing microtubule tips, which were detected close to actin coats on fused vesicles (Fig. 8B) or moved close to actin coats (Fig. 8C). CLIP170 localisation was quantified by counting the number of CLIP170-mEmerald labelled microtubule tips on the region of interest around the actin coat at different time points after fusion. Quantification showed that CLIP170-mEmerald-stained microtubule tips were associated with 40.7% to 79.2% of fusing vesicles during 180 s after vesicle fusion (Fig. 8D). Proximity between IQGAP1-GFP and actin coats as well as proximity between CLIP170-mEmerald and actin coats suggests that these proteins might enable interaction of microtubules and actin on fused vesicles.

## Discussion

Microtubules and actin interact during cell migration, cytokinesis, neurite outgrowth and phagocytosis; however, it is not known if they also interact during secretion. Here we show that microtubules were located close to actin coats on fused secretory vesicles and stayed close to the coats during their compression. Treatment with microtubule polymerisation inhibitors colchicine and nocodazole altered kinetics of actin coat formation and augmented actin polymerisation on fused vesicles, suggesting a functional link between both cytoskeletal networks. The connection between microtubules and actin could be provided by actin and microtubule crosslinking proteins such as IQGAP1. Down-regulation of IQGAP1 resulted in weaker actin polymerisation on fused vesicles.

The proximity of microtubules and compressing actin coats as well as localization of EB1-GFP and CLIP170-mEmerald close to actin coats suggest a dynamic interaction and a functional connection between both networks. The estimated extent of actin polymerisation on actin coats was consistently higher in cells treated with microtubule disrupting agents (colchicine or nocodazole) then in control cells. Previously it was shown that microtubule depolymerisation stimulates Rho GTPase activity^39,40,49^, which leads to enhanced polymerisation of actin filaments^35,38^ via activation of formin nucleation factor mDia^37^. Our previous work showed that actin coat formation in ATII cells depends on Rho and formins^12,13^, therefore it is likely that this process was affected by disruption of the microtubule network.

We also observed that the peak fluorescence of actin coats during their formation was reached more slowly in cells treated with colchicine or nocodazole. A possible explanation could be that treatment with microtubule depolymerising agents resulted in increased actin polymerisation and more time was required to reach the peak of actin accumulation. This could result in the observed difference in the relative kinetics of actin coat formation.

Next, we searched for a molecular mechanism that could link microtubule remodelling to actin polymerisation. We found that microtubule and actin cross-linking factor IQGAP1, which binds small GTPases and regulates actin polymerisation via formin mDia, translocates to the membrane of fused secretory vesicles. Interestingly, IQGAP1 translocation took place even when actin coating was inhibited by latrunculin. IQGAP1 was therefore most likely recruited to the membrane of fused vesicles by factors other than actin. A potential candidate for directed IQGAP1 translocation is active Rho, which is present at the fused vesicle membrane before actin coat formation^13^, and binds IQGAP1^65^. However, inhibition of Rho GTPases by B-toxin or by Rho inhibitor did not hinder IQGAP1-GFP translocation to fused vesicles, which suggests that IQGAP1 recruitment to fused secretory vesicles did not depend on small GTPases. A similar observation was made by Brandt *et al*.^51^ who found that inactivation of Rho did not affect IQGAP1 translocation to the leading edge of migratory cells. However, IQGAP1 was reported to bind directly or indirectly to more than 90 proteins^66^ and it is likely that it was recruited to the fused vesicles by the factors not investigated in this study. Actin assembly on fused vesicles is thought to be triggered by membrane mixing and diffusion of key components from the plasma membrane to the vesicle membrane^67^. IQGAP is involved in Akt signalling and was shown to have a binding site for phosphoinositides^68^. Therefore, phosphoinositides or associated proteins are possible candidates for IQGAP1 recruitment.

After investigating IQGAP1 translocation, we studied its function during actin coat formation and compression. IQGAP1 silencing resulted in reduced actin-GFP fluorescence on actin coats. IQGAP1 promotes actin nucleation via N-WASP and Arp2/3^50^ and is also necessary for proper localisation of formin nucleation factor Dia1^51^. It is therefore possible that IQGAP1 provides a scaffold for nucleation of branched actin filaments via Arp2/3 as well as nucleation of unbranched filaments via formins. This may help to explain previous findings that suggest participation of formins^10,12^, as well as N-WASP and Arp2/3^56,67^ in formation of actin coats.

IQGAP1 associates with microtubules via CLIP170 and we detected CLIP170-mEmerald on microtubule tips close to compressing actin coats. Interaction between CLIP170 on microtubules and IQGAP1 on actin coats could contribute to cross-talk between actin and microtubules. Previous *in vitro* experiments demonstrated that binding of CLIP170 to formin mDia1 accelerated actin polymerisation and protected actin barbed ends^69^. This suggests that microtubule growing tips can influence actin dynamics during actin coat compression.

To conclude, the results of this study indicate an interaction between microtubules and actin during the post-fusion phase of exocytosis. This interaction can be mediated by microtubule and actin cross-linking protein IQGAP1. Other molecular players that may provide either structural links between both cytoskeletal networks or participate in signaling cascades affecting their dynamics remain to be established. Moreover, interaction between microtubules and actin could also be present in other stages of secretion, such as vesicle transport and cortical actin remodeling.

## Material and Methods

### Cell isolation and transfection

Primary ATII cells were isolated from the lungs of male Sprague-Dawley rats according to the procedure of Dobbs *et al*.^70^ with minor modifications^71^. All experiments in this study were approved by the Regierungspräsidium Tübingen, Germany. All methods were performed in accordance with the relevant guidelines and regulations. Isolated cells were seeded on 8-well chamber slides (Ibidi, München, Germany) and cultured in MucilAir medium (Epithelix, Geneva, Switzerland). ATII cells were transfected with EB1-GFP, tubulin-mRuby, IQGAP1-GFP, CLIP170-mEmerald or GFP by electroporation of expression constructs with 4D-Nucleofector (Lonza, Köln, Germany) using Amaxa basic nucleofector kit for primary mammalian epithelial cells. Actin-GFP, actin-DsRed and lifeact-GFP were introduced in cells by adenoviral vectors, whereas Viromer Blue transfection system (Lipocalyx, Halle, Germany) was used to transfect ATII cells with siRNA according to manufacturer’s instructions.

### Plasmids, adenoviral vectors and siRNA

Plasmid for expression of tubulin-mRuby (pcDNA3-mRuby-αTubulin) was described previously^72^ and was a kind gift from Franz Oswald (Ulm University, Germany). Plasmid pGFP-EB1 was a gift from Lynne Cassimeris (Addgene plasmid # 17234)^73^. Plasmid pEGFP-IQGAP1 was a gift from David Sacks (Addgene plasmid # 30112)^74^. Plasmid mEmerald-CLIP170-C-18 was a gift from Michael Davidson (Addgene plasmid # 54043). Plasmid pmax GFP™ was from Lonza (Germany). Adenovirus vectors expressing actin-GFP and actin-DsRed were described previously^12,54^. Lifeact-GFP was purchased from Ibidi (München, Germany). IQGAP1 expression was inhibited by double-stranded 21-mer RNA strands (IQGAP1 siRNA sense sequence CGAGGAACAUGAGCGGAUUtt, antisense sequence: AAUCCGCUCAUGUUCCUCGtg). IQGAP1 siRNAs as well as siRNA negative control were purchased from Silencer Select Pre-designed siRNA from Ambion (Thermo Fisher Scientific, Braunschweig, Germany). For microscopy experiments siRNA was fluorescently labelled using Silencer siRNA labelling kit (Life Technologies, Darmstadt, Germany) to identify the transfected cells.

### Experimental conditions

Experiments with ATII cells were performed in bath solution (in mM: 140 NaCl, 5 KCl, 1 MgCl_2_, 2 CaCl_2_, 5 glucose, 10 Hepes; pH 7.4). Cells were stimulated for secretion with 100 μM ATP (Sigma, Schnelldorf, Germany). Fusions were detected by staining secretory vesicles with Lysotracker Blue (100 nM) or LysoTracker Red (10 nM) for 20 min (Molecular Probes, Life technologies, Darmstadt, Germany). LysoTracker dyes accumulate in LBs and rapidly diffuse out of the vesicle after fusion^52^. Microtubule polymerization was inhibited by colchicine (50 µM for 3 h) or nocodazole (60 µM for 30 min), and actin polymerization was inhibited by latrunculin B (10 µM in the experimental bath solution), all from Sigma Aldrich (Steinheim, Germany). Small GTPases were inhibited by B-toxin (300 ng/ml, for 24 h; Merck Millipore, Billerica, USA) and Rho-Inhibitor (2 μg/ml, 12 to 24 h, Cytoskeleton, Inc., Denver, USA).

### Semi-quantitative RT-PCR

We performed RT-PCR using 0.2–0.3 µg RNA, SuperScript VILO synthesis kit and QuantiTect primer assays (Quiagen, Hilden, Germany) on a realplex2 mastercycler (Eppendorf, Hamburg, Germany) as described in detail previously^13^.

### Immunoblotting

ATII cells (2 × 10^6^) were washed twice in PBS buffer and suspended in lysis buffer. For detection of IQGAP1 SDS-PAGE was performed using NuPAGE LDS Sample Buffer, NuPAGE MES SDS Running Buffer and NuPAGE Novex 4% to 12% Bis-Tris Protein Gel. Electroblotting was performed with iBLOT under constant voltage of 200 V for 10 min using nitrocellulose membranes. Immunodetection was performed using Western Breeze Chromogenic Kit. Consumables for immunoblotting were purchased from Thermo Fisher Scientific (Braunschweig, Germany). Primary antibody anti-IQGAP1 (ab110203) was purchased from Abcam (Cambridge, UK) and used at a dilution of 1:1000.

### Electron microscopy

ATII cells were seeded on glow discharged, carbon coated sapphire discs (3 mm in diameter, 160 µm thick, Engineering Office M. Wohlwend GmbH, Sennwald, Switzerland). After 48 hours (37 °C, 5% CO2) the cells were high pressure frozen (Wohlwend HPF Compact 01 high-pressure freezer; Engineering Office M. Wohlwend GmbH, Sennwald, Switzerland) as described by Buser and Walther^75^ and freeze substituted with substitution medium (glutaraldehyde 3%, uranyl acetate 0.1%, water 1.2% in acetone). Temperature was raised from 183 K to 273 K in 18 h. Samples were embedded in epon and 70 nm sections were cut on a Ultracut UCT (Leica). Images were acquired with a JEOL-1400 (JEOL, Tokyo, Japan) at 120 kV acceleration voltage.

### Immunofluorescence

Primary antibodies were purchased from Abcam (Cambridge, UK) and were used at following dilutions: β-tubulin (ab6064; 1:200), ABCa3 (ab24751; 1:300) and IQGAP1 (ab110203; 1:200). Fluorescently labelled secondary antibodies and alexa fluor 568 phalloidin were purchased from Molecular Probes (Invitrogen, Carlsbad, USA) and used at dilution of 1:400. Immunofluorescence was performed as described previously^16^.

### Fluorescence imaging

All fluorescence imaging experiments were performed on iMic digital microscope (Till Photonics, Gräfelfing, Germany). Images were acquired at 1 frame per 1.5 s for 5 min using iMic Online Analysis software (Till Photonics, Gräfelfing, Germany), 40x oil objective and multipass filter with dichroic mirror 405/488/561/640 nm; emission filter 446/523/600/677–25 nm and excitation filters 390/40 nm, 482/18 nm, 563/9 nm, and 640/14 nm.

### Image analysis and data presentation

Images were analysed using Fiji (NIH, Bethesda, United States). A circular region of interest was set around the fusing LB on the image sequence to measure the time of LB fusion (LysoTracker channel) or the translocation of fluorescently labelled constructs to fusing vesicles^12,13^. To measure actin coat fluorescence intensity, a ring-shaped region of interest was drawn on the site of circular actin coats (Fig. 5B, insert). Mean fluorescence intensity was measured on fully formed actin coats before their compression and on the region of interest of the same size in cell cytoplasm. Fluorescence intensity of actin coat (a) was normalized to the fluorescence of cell cytoplasm (c) by calculating ratio ([a–c]/c) as described previously for protein accumulation on phagocytic cups^76,77^. Actin coat compression after fusion was analysed by measuring the vesicle diameter at indicated time points after fusion. Colour coding of EB1-GFP image sequence was performed with Temporal-Color Code function in Fiji after the first image was subtracted from image stack to reduce background as described before^78^. The vesicle fusions were measured from at least 4 independent experiments from at least 3 cell isolations. D’Agostino and Pearson omnibus normality test was used for estimation of data distribution. Images were corrected for contrast and brightness in Photoshop CC (Adobe, San Jose, USA), which was also used to create overlays and colour images. Microsoft Excel and GraphPad Prism 5–7 (GraphPad Software, La Jolla, USA) were used for statistics and graph design. Unless otherwise stated all data are presented as mean ± SEM (standard error of the mean).

## Supplementary information

Supplemental movie 1

Supplemental movie 2

Supplemental information

## Data Availability

The data generated and analysed during the current study are included in this article and detailed datasets are available on request from the corresponding author.
