# Supplementary material for: Interaction of microtubules and actin during the post-fusion phase of exocytosis
Source: Sci Rep. 2019 Aug 19;9:11973. doi: 10.1038/s41598-019-47741-0 (PMC6700138; doi:10.1038/s41598-019-47741-0)

Interaction of microtubules and actin during the post-fusion phase of exocytosis

M. Tabitha Müller, Rebekka Schempp, Anngrit Lutz, Tatiana Felder, Edward Felder, Pika Miklavc

Supplementary Figure Legends

Supplementary figure 1: Fluorescence intensity profiles of Tubulin Tracker Green and actin-DsRed measured across the fusing vesicle.

Examples of fluorescence intensity profiles for individual fusing vesicles with actin coats that fully compressed (A-C, F) and vesicles where actin coats only partially compressed during the experiment (D, E, G). Numbers above the lines indicate time in seconds. Time = 0 is the last frame before vesicle fusion with the plasma membrane indicated by LTB fluorescence decrease.

Supplementary figure 2: Fluorescence intensity profiles of GFP and actin-DsRed measured across the fusing vesicle.

A-E) Examples of fluorescence intensity profiles for individual fusing vesicles in cells co-transfected with control GFP plasmid and with actin-DsRed. Numbers above the lines indicate time in seconds. Time = 0 is the last frame before vesicle fusion with the plasma membrane indicated by LTB fluorescence decrease.

Supplementary figure 3: Microtubule translocation during vesicle compression in cells co-transfected with actin-GFP and tubulin-mRuby.

A) ATII cells were co-transfected with actin-GFP and tubulin-mRuby. The vesicles were labelled with LTB. Arrow indicates a fusing secretory vesicle enlarged in B. Scale bar: 10 µm.

B) Time lapse image sequence of a fusing vesicle in ATII cell transfected with tubulin-mRuby and actin-GFP and stained with LTB. Numbers indicate time in seconds. Time = 0 is the last frame before vesicle fusion with the plasma membrane. Fusion was followed by formation and partial compression of the actin coat. Microtubules translocated to the space that was occupied by the vesicle before actin coat compression. Yellow dashed line was used

to create the fluorescence profiles on C and the white rectangle was used to create kymographs on D. Scale bar: 2 µm.
C) Fluorescence intensity profiles of tubulin-mRuby (green) and actin-GFP (red) measured across the fusing vesicle (dashed yellow line on B). Numbers indicate the time after fusion.
D) Kymographs of tubulin-mRuby, actin-GFP and LTB fluorescence were constructed using a region of interest on B (dashed white rectangle). Actin coat compression was accompanied by translocation of microtubules. Scale bars: 10 s and 2 µm.
E) Compression of fused vesicles in cells transfected with actin-GFP and tubulin-mRuby. Compression was measured as a decrease in vesicle diameter in actin-GFP (red) and in tubulin-mRuby image sequence (green). The lines indicate mean +/- SEM. N=15 vesicles from 10 independent experiments and 3 cell isolations.

Supplementary figure 4: EB1-labelled microtubule tips localize close to compressing actin coats
A) ATII cells were transfected with EB1-GFP, which localizes to microtubule tips. Tracking EB1-GFP over time generated a time projection of microtubule tip movement. Overlay shows time projection superimposed on the fluorescence image. Colour code for time projection over 200 frames of the experiment is on the right. 1 frame = 1.5 s. Scale bar = 10 µm.
B) ATII cells were co-transfected with EB1-GFP and actin-DsRed and stained with LTB. The region with fusing vesicles (arrow) is enlarged in C. Scale bar = 10 µm.
C) Image sequence shows EB1-GFP, actin-DsRed and LTB fluorescence for three fusing vesicles (arrows). EB1-GFP fluorescence intensity increased around compressing vesicle (arrowhead). Time stamps indicate time in seconds. Time = 0 is the last time frame before fusion. Scale bar: 2 µm.
D) Time projection of EB1-GFP for the image shown in B. The region with fusing vesicles (arrow) is enlarged in E. The colour code for tracking over 200 frames of the experiment is below the image (1.5 s / frame). Scale bar: 10 µm.
E) Time projection (Time p.) of microtubule movement, actin-DsRed fluorescence (actin) and overlay of both for the region with three fusing vesicles from D. Arrows point at three actin coats. The dashed white rectangle shows the growth of one microtubule along the actin coat of the bottom vesicle and was used to create kymographs on F. Scale bar: 2 µm.
F) Kymographs of EB1-GFP and actin-DsRed were constructed using a region of interest on E. Black arrowheads mark the microtubule growth and the white arrowhead marks the formation of the actin coat. Scale bars: 10 s and 2 µm.

Supplementary figure 5: Treatment of ATII cells with colchicine or nocodazole caused microtubule depolymerisation.

ATII cells were treated with either colchicine (50 µM for 3 h) or nocodazole (60 µM for 30 min) and subsequently immunolabelled with anti-β-tubulin antibody. Treated cells show the absence of microtubule network. Scale bar: 10 µm.

Supplementary figure 6: Actin coats in cells immunostained with alexa fluor 568 phalloidin were identified by staining the vesicles with anti-ABCa3 antibody.

ATII cells were treated with colchicine or nocodazole, stimulated for secretion with ATP, fixed and immunostained with alexa fluor 568 phalloidin and anti-ABCa3 antibody. Actin rings on phalloidin images were confirmed as actin coats by comparison with ABCa3 staining (arrows).

Supplementary figure 7: Full-length immunoblotting gel for IQGAP1 detection.

A) Western blot with α-IQGAP1 antibody with freshly isolated ATII cells (d0), after 1 day of culture (d1) and after 2 days of culture (d2). M = Molecular weight marker.

B) The same gel in Ponceau S staining as control for equal loading of the lanes.

Supplementary figure 8: Full-length immunoblotting gel to detect the effect of IQGAP1-silencing siRNA

A) Immunoblotting with α-IQGAP1 antibody three days after cell isolation and transfection with IQGAP1-silencing siRNA (si) or control siRNA (c). Ut: untreated control. M = Molecular weight marker.

B) The same gel in Ponceau S staining as control for equal loading of the lanes.

Supplementary Movie Legends

Supplementary movie 1: EB1-GFP-labelled microtubule growing ends converged on compressed actin coats

ATII cells were co-transfected with EB1-GFP (green) and actin-DsRed (red) and stained with LTB (blue). EB1-GFP localized to microtubule tips enabling visualization of growing microtubules. Fused vesicles were detected by decrease in LTB fluorescence and acquisition

of actin coats. EB1-GFP fluorescence intensity increased on the compressed actin coat (arrow). Time stamps indicate time in seconds.

Supplementary movie 2: CLIP170-mEmerald-labelled microtubule growing ends converged on compressed actin coats

Image sequence showing part of ATII cell co-transfected with CLIP170-mEmerald (green) and actin DsRed (red). CLIP170-mEmerald-labelled microtubule growing ends were detected close to the fusing vesicles and on site of fully compressed actin coats (arrows). Time stamps indicate time in seconds.

Supplementary figure 1

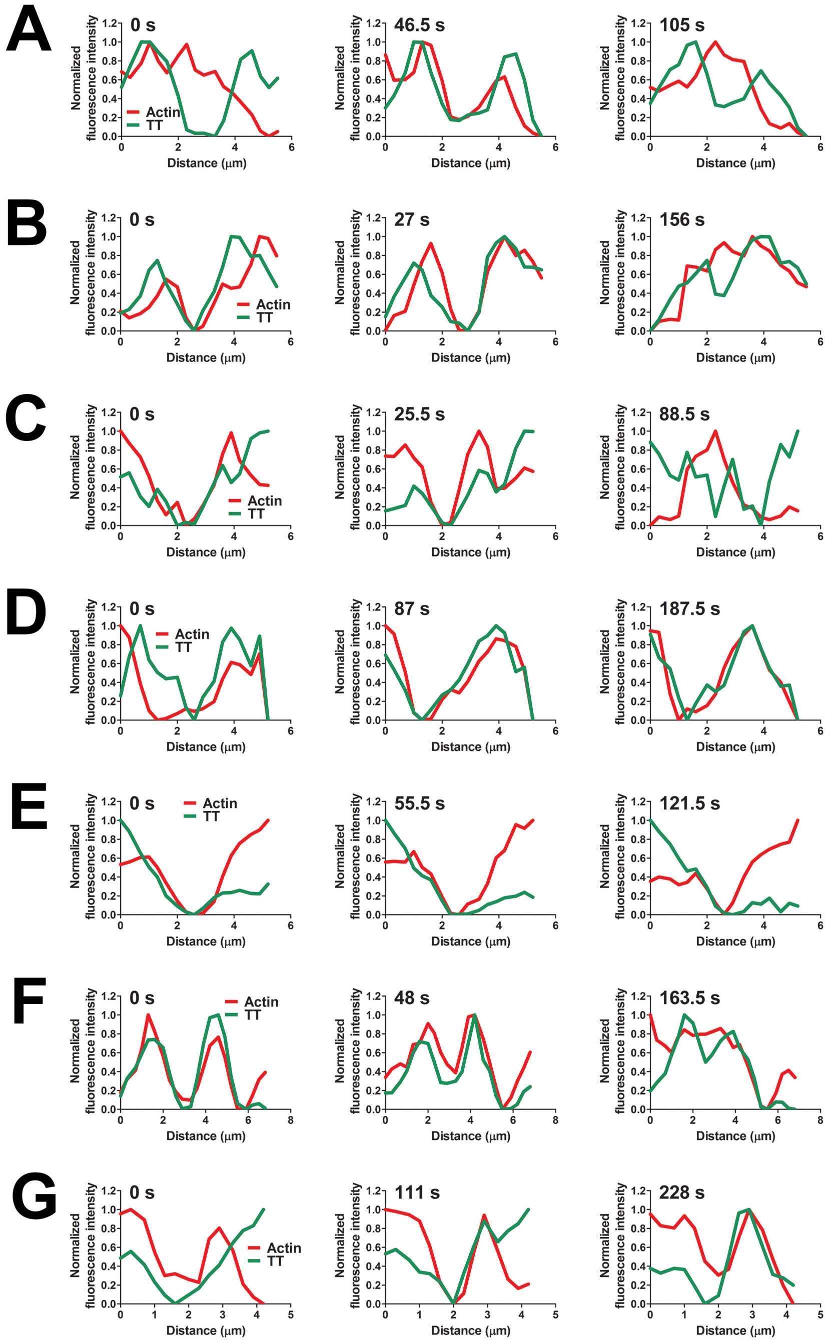

Supplementary figure 2

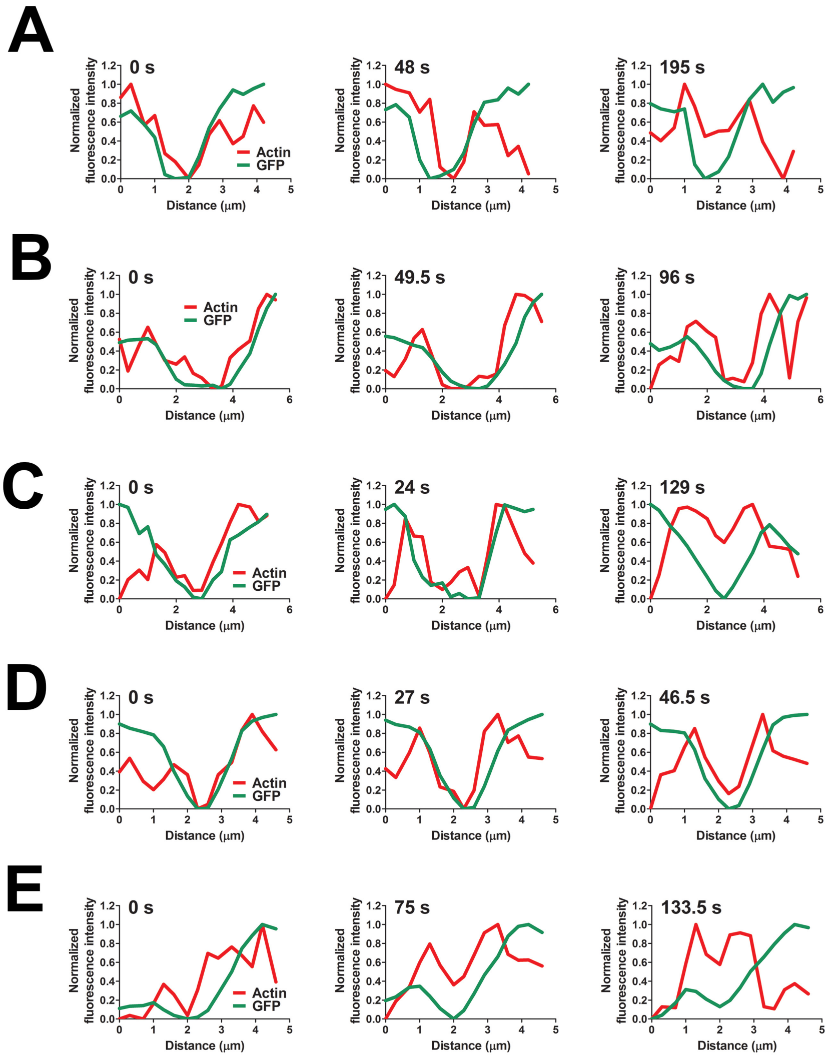

Supplementary Figure 3

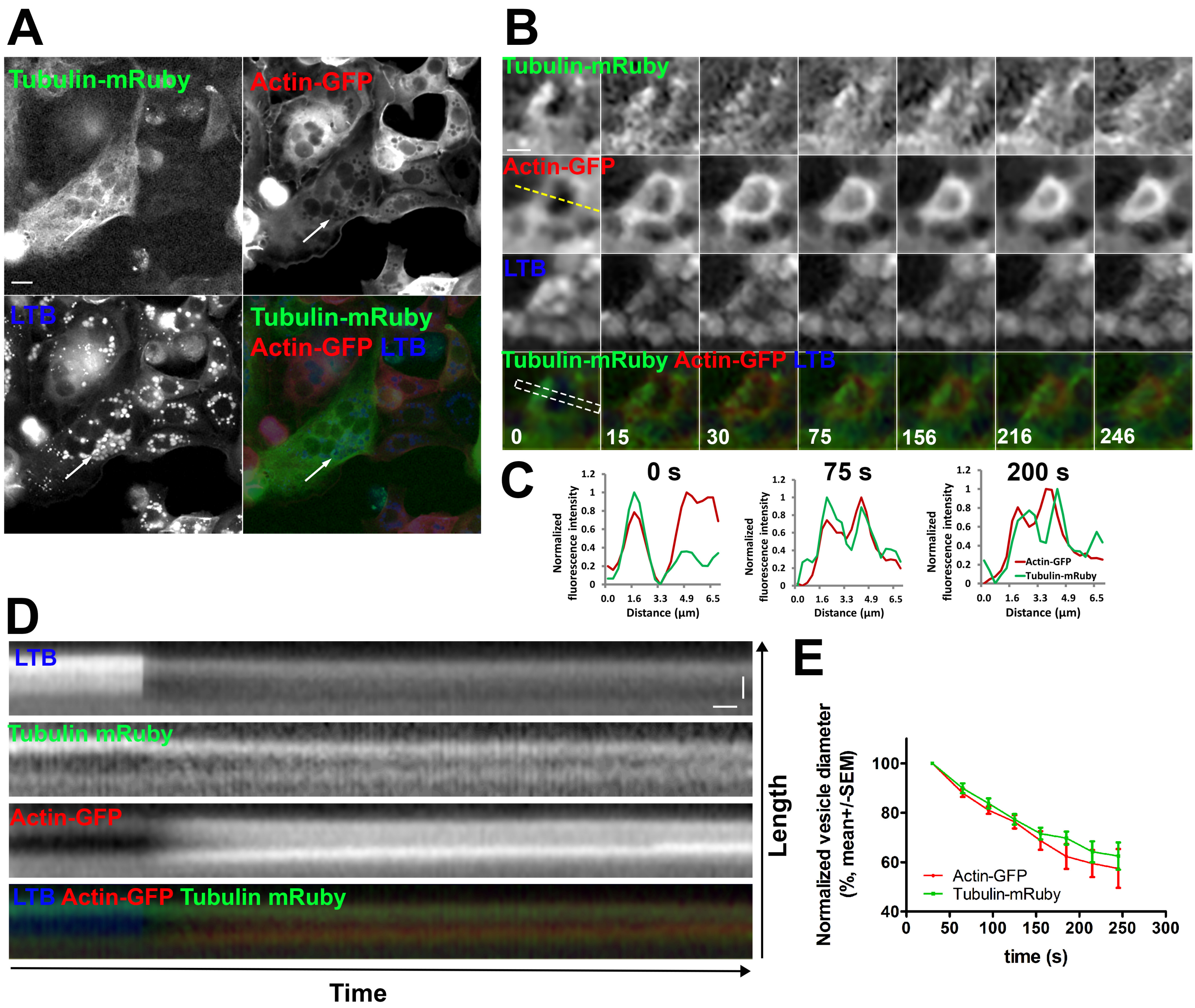

Supplementary Figure 4

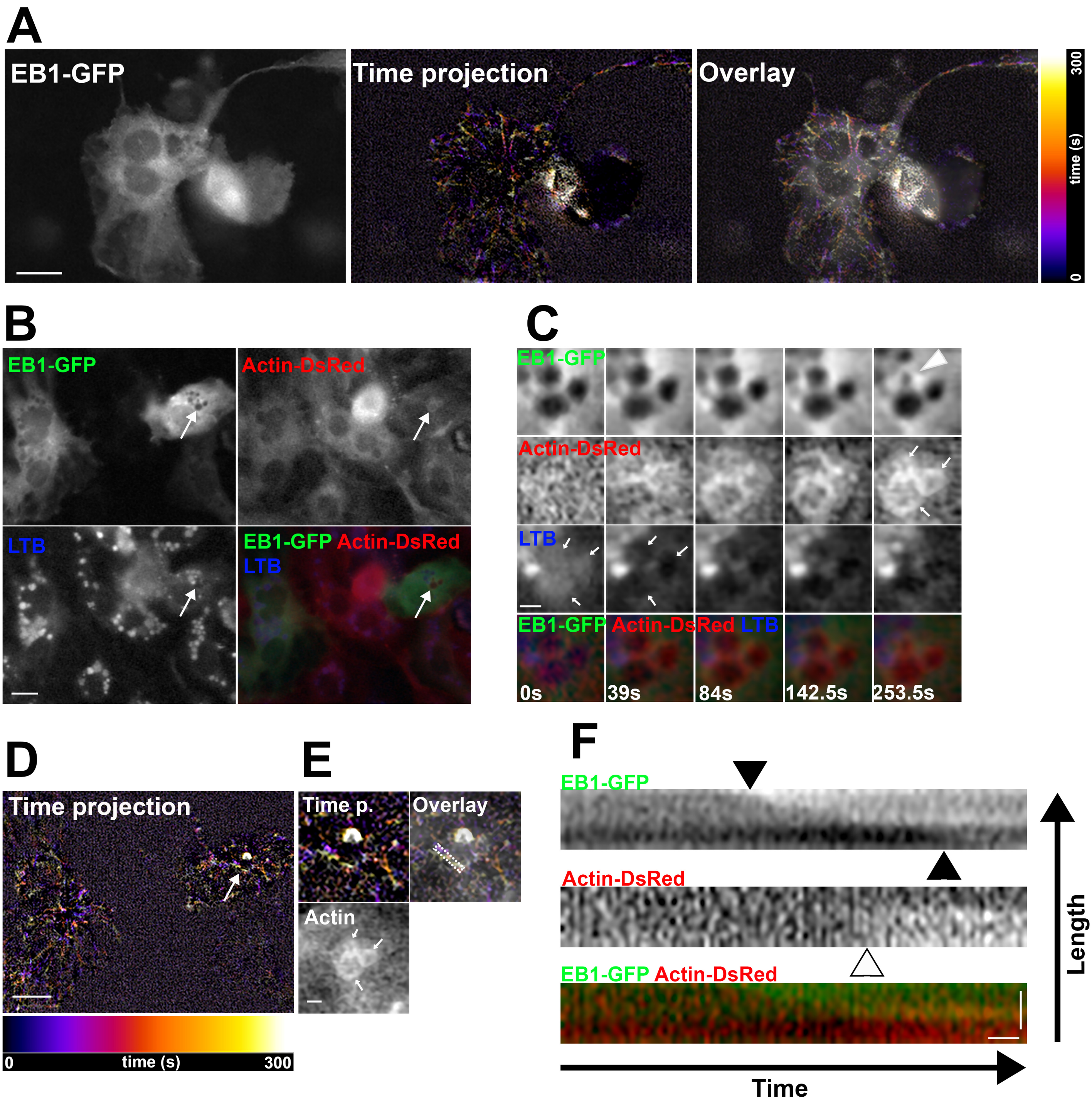

Supplementary figure 5

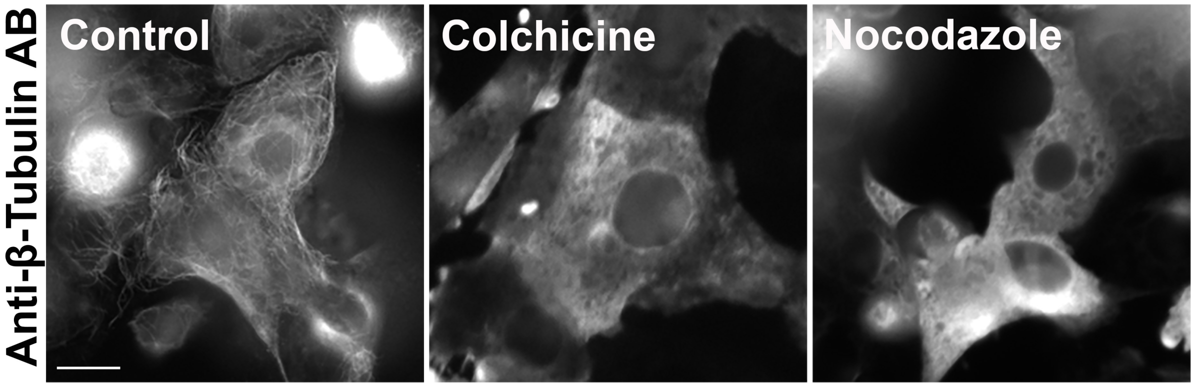

Supplementary figure 6

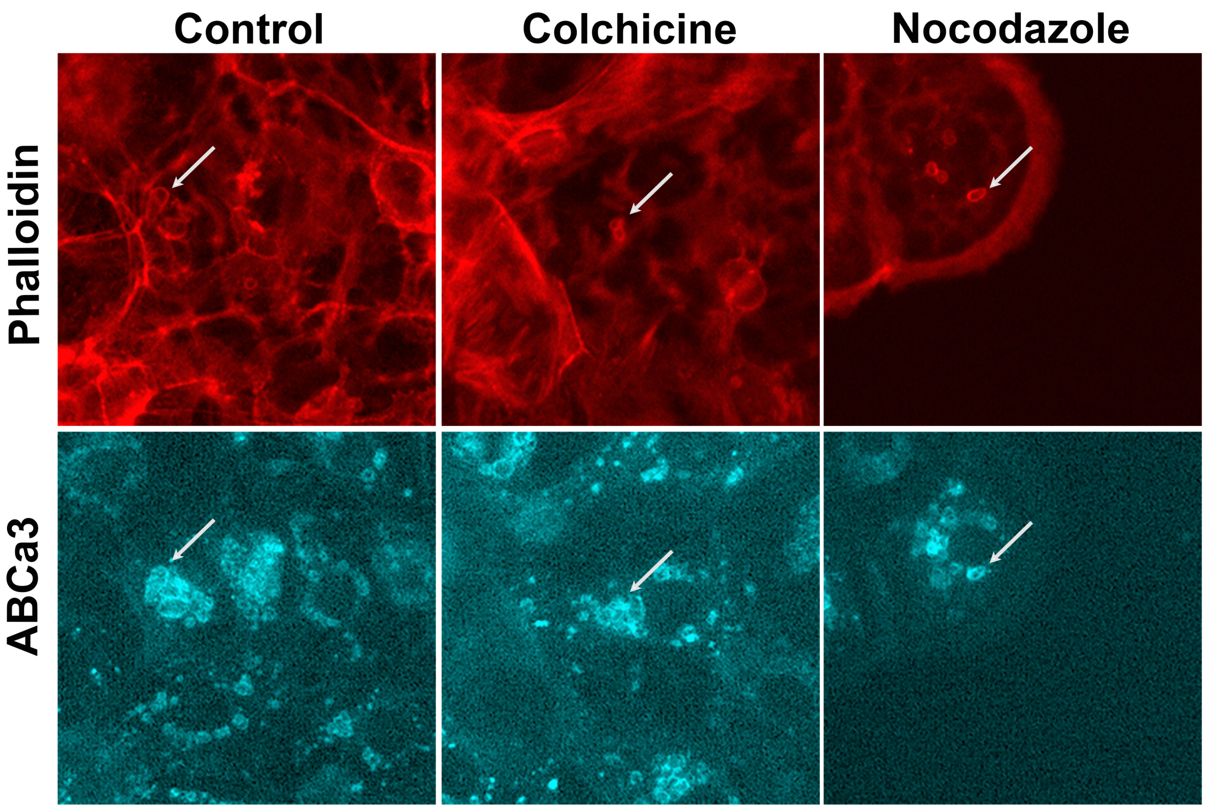

Supplementary figure 7

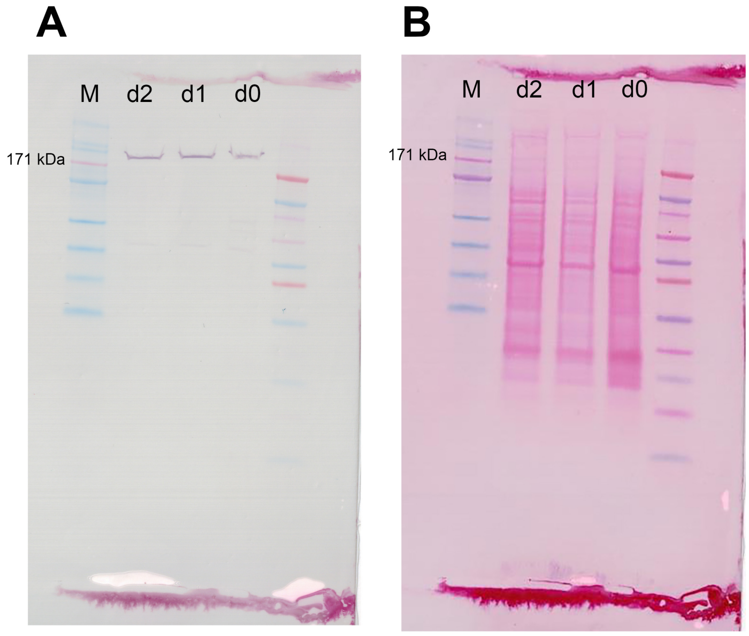

Supplementary figure 8

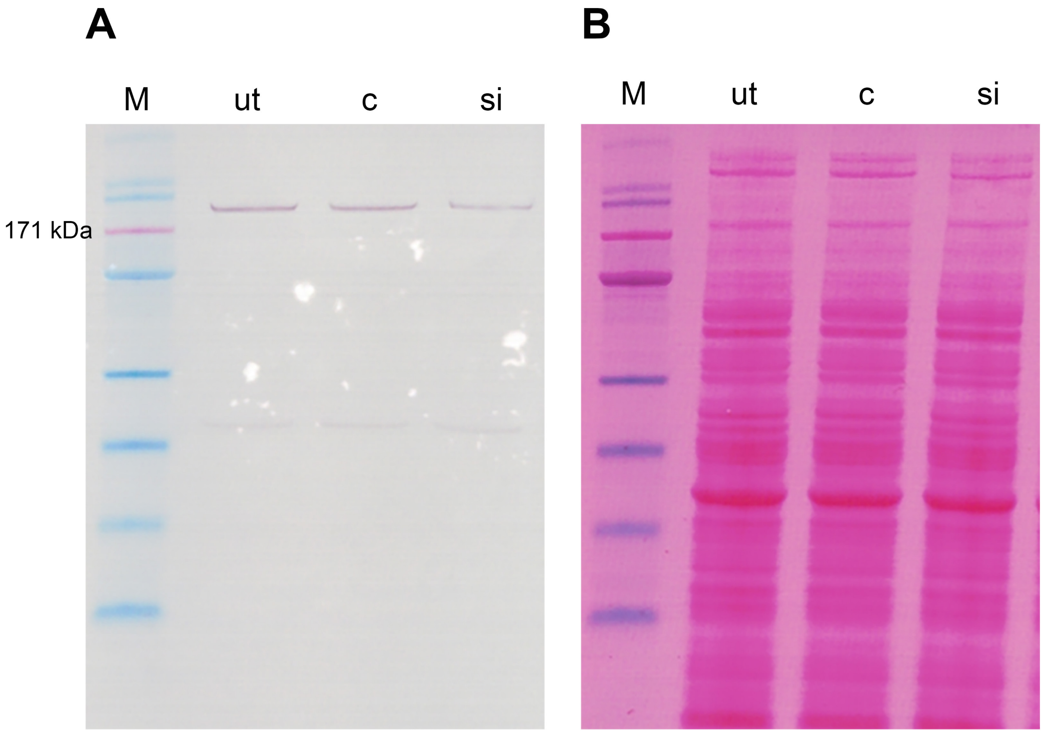

Supplement: Supplementary file 3 — Supplemental information [file 41598_2019_47741_MOESM3_ESM.pdf]
